# Acculturation and suicide-related risk in ethnoracially minoritized youth in the US: a scoping review and content analysis of the empirical evidence

**DOI:** 10.1007/s00127-023-02494-0

**Published:** 2023-06-04

**Authors:** Lillian Polanco-Roman, Chantel T. Ebrahimi, Katherine S. W. Mafnas, Carolina Hausmann-Stabile, Alan Meca, Silvia L. Mazzula, Cristiane S. Duarte, Roberto Lewis-Fernández

**Affiliations:** 1 The New School, Department of Psychology, 80 Fifth Avenue, Room 617, New York, NY 10011 USA; 2 Department of Psychiatry, New York State Psychiatric Institute, Columbia University, New York, NY USA; 3 Graduate School of Social Work and Social Research, Bryn Mawr College, Bryn Mawr, USA; 4 Department of Psychology, University of Texas at San Antonio, San Antonio, USA; 5 Department of Psychology, John Jay College for Criminal Justice, CUNY, New York, NY USA

**Keywords:** Suicidal ideation, Suicide attempt, Adolescents, Acculturation, Ethnicity, Race

## Abstract

**Purpose:**

Among Asian-American/Pacific Islander, Hispanic/Latinx, and Black youth, the US born have higher risk of suicidal thoughts and behaviors (attempts and death-by-suicide) than first-generation migrants. Research has focused on the role of acculturation, defined as the sociocultural and psychological adaptations from navigating multiple cultural environments.

**Methods:**

Using content analysis, we conducted a scoping review on acculturation-related experiences and suicide-related risk in Asian-American/Pacific Islander, Hispanic/Latinx, and Black youth (henceforth described as “ethnoracially minoritized adolescents”), identifying 27 empirical articles in 2005–2022.

**Results:**

Findings were mixed: 19 articles found a positive association between acculturation and higher risk for suicide ideation and attempts, namely when assessed as acculturative stress; 3 articles a negative association; and 5 articles no association. Most of the research, however, was cross-sectional, largely focused on Hispanic/Latinx youth, relied on demographic variables or acculturation-related constructs as proxies for acculturation, used single-item assessments for suicide risk, and employed non-random sampling strategies. Although few articles discussed the role of gender, none discussed the intersections of race, sexual orientation, or other social identities on acculturation.

**Conclusion:**

Without a more developmental approach and systematic application of an intersectional research framework that accounts for racialized experiences, the mechanisms by which acculturation may influence the risk of suicidal thoughts and behavior remain unclear, resulting in a dearth of culturally responsive suicide-prevention strategies among migrant and ethnoracially minoritized youth.

## Introduction

In recent decades, youth suicide deaths [1] and suicidal behaviors [2, 3] in the United States show a disproportionate increase among Asian-American/Pacific Islander, Hispanic/Latinx, and Black youth (henceforth described as “ethnoracially minoritized youth”). Further, early studies identified higher rates of suicidal thoughts and behaviors in US-born ethnoracially minoritized youth compared to their foreign-born migrant peers [4–7]. This is especially concerning as the US youth population becomes increasingly ethnoracially diverse, largely due to the influx of migrants from the Global South (Latin America and the Caribbean, Asia, and Africa) [8]. Sociocultural context is critical for understanding suicide-related risk as evidenced by prevalence differences by race, ethnicity, and immigrant background [9]. Nevertheless, it is largely overlooked in the suicide literature [10] and contemporary suicide frameworks [11, 12].

Acculturation—the process of sociocultural and psychological adaptations resulting from navigating multiple cultural environments [13]—is salient in the lives of many youths in the US and warrants further attention. To provide more culturally responsive suicide-prevention strategies, a better understanding of how acculturation can influence youth suicide-related risk is critical. Through content analyses—examination of text data used to systematically quantify and identify patterns to interpret meaning[14]—this scoping review synthesizes the existing empirical evidence on the potential role of acculturation and acculturation-related factors (e.g., acculturative stress) in suicide-related risk among ethnoracially minoritized US-based youth. We discuss public health and clinical implications, highlight important gaps, and provide future directions in this area of study.

Adolescence—the developmental stage between childhood and adulthood—is a high-risk period for preventable deaths involving health-risk activities [15] such as suicidal behaviors, whose onset and prevalence peak during this time [16]. Adolescence is also characterized by young people’s growing awareness of how their identity is shaped by social categories and group memberships, including their race, ethnicity, and immigration status [17]. This process is complicated for ethnoracially minoritized youth because they are additionally tasked with navigating their marginalized identities within a specific historical, socio-political US context of oppressive racial hierarchies, yielding unique experiences and challenges that may impact mental health outcomes [18–20]. Thus, acculturative experiences may be especially relevant for clarifying suicide-related risk in migrant and ethnoracially minoritized youth.

Contemporary models conceptualize acculturation as changes in cultural orientation along at least two dimensions (i.e., heritage, mainstream) resulting from reconciling these changes along various domains: practices, values, and identity [13]. Within this bidimensional, multiple-domain framework, mainstream culture acquisition does not imply that individuals discard their own cultural heritage. For some individuals, acculturation may engender specific challenges and barriers in accessing societal resources (e.g., employment, health insurance coverage, and education) that directly or indirectly impact access to safe living conditions and quality health care services [21]. Consequently, research increasingly suggests that acculturative processes can play a critical role in health outcomes. However, findings to date vary across ethnoracial groups, are largely focused on migrants, are over-represented by adult samples, and rarely examine suicide-related outcomes [22]. Although largely studied among migrants, acculturative processes have also been identified in non-migrant, non-dominant groups, such as Native American/American Indian/Alaskan Native [23] and African-American populations [24]. These groups are also subjected to political violence and oppression and often navigate between their heritage cultures and the dominant Euro-American culture due to the legacy of colonization and slavery. For migrants, acculturation often involves navigating their relationship to other minoritized cultural communities as well as to the dominant Euro-American cultural group, magnifying the complexity of the process [25].

Research on how this multifaceted acculturation process may shape youth suicide risk remains scarce and has produced mixed findings [26]. This literature has also largely relied on small, geographically specific samples of largely Mexican-descent populations and has been outpaced by the changing demographics of US youth [26]. Recent literature reviews on suicide risk in migrant and ethnoracially minoritized groups found some evidence for acculturation-related factors, particularly acculturative stress [27–29]—the subjective distress from navigating a novel cultural environment [30, 31]. Acculturative stress can involve pressures to assimilate, to oppose acculturation, and experiences of ethnoracial discrimination, intergenerational conflict, or language and other cultural barriers [9]. Moreover, concerns about the conceptualization and assessment of acculturation have also hampered this research, including lack of consensus on the operationalization of acculturation and the overreliance on demographic variables as proxies for acculturation indices [30, 32]. Scholars propose that the operationalization of acculturation may be refined by distinguishing the overall acculturative process, which may not be inherently harmful to health, from acculturation-related experiences that include challenges and stressors that are more likely to negatively impact health, such as acculturative stress.

Acculturation frameworks have also been criticized for their singular focus on individual-level processes, which overlook the impact of structural factors derived from societal systems rooted in racism and the legacies of colonization and slavery, resulting in inequitable access to societal resources. These structural factors inevitably shape the acculturative process [33, 34], particularly among ethnoracially minoritized youth [35], by intersecting youths’ migration status with social positionality (e.g., race, ethnicity, and sexual orientation) that affect how people navigate cultural environments and how this navigation impacts health [33]. For example, national data on adolescent health risk behaviors show that Black, and Hispanic/Latina girls (compared to boys of the same groups) and LGBTQ-identifying youth from ethnoracially minoritized backgrounds (compared to White LGBTQ youth) are at particularly elevated risk for suicidal behaviors [36]. Although there is substantial empirical evidence [37, 38] showing that racism is a principal driver of racial and ethnic disparities in health outcomes, and that it constitutes a social determinant of health independently of poverty, the role of race and racism remains overlooked in contemporary acculturation frameworks and youth suicide research.

This scoping review aims to determine the extent, quality, and key findings of the empirical evidence on the role of acculturation and acculturation-related experiences in shaping suicide-related risk among ethnoracially minoritized youth in the United States. We highlight critical gaps in the literature and provide recommendations to help guide future research on culturally responsive suicide-prevention strategies.

## Methods

### Search strategy

This review follows the Preferred Reporting Items for Systematic Reviews and Meta-analyses extension for Scoping Reviews (PRISMA-ScR) as outlined in Tricco and colleagues’ [39] scoping review protocol. We chose 2005 as the starting point because that was when the first acculturation and youth suicide framework, the eco-developmental model of suicidal behaviors in Latina adolescents, was introduced by Zayas and colleagues [40]. An online database search was conducted from 01/01/2005 to 03/31/2022 using the following databases in psychology, social work, nursing, psychiatry, public health, and general social sciences: Google Scholar, ProQuest, PsycINFO, and PubMed. Only peer-reviewed published articles in English were selected. The search terms used were (acculturat* OR immigrat*) AND suicid* AND (adolesce* OR teen*). This search yielded *N* = 533 articles. After we excluded review papers, case studies, commentaries or letters to the editors, conceptual papers, duplicates, and random hits (i.e., pieces not meeting our selection criteria, such as dissertations, theses, books, book chapters, or non-English-language publications), the remaining peer-reviewed empirical research articles were selected for further screening.

### Inclusion criteria

The full texts of these peer-reviewed articles were evaluated using the following eligibility criteria:

- Comprised original research with ethnoracially minoritized adolescents (ages 11–20) in the US, inclusive of Alaska/Hawaii/US territories
- Included data on ethnoracially minoritized adolescents presented separately from the non-Latinx White adolescents subsample
- Reported suicidal ideation, planning, attempts, and/or deaths, excluding articles examining non-suicidal self-harm behaviors without intent to die, regardless of informant (e.g., youth, parents, and teachers)
- And examined the relationship between acculturation, acculturation proxies via demographic variables (e.g., generational status), and/or acculturation-related constructs (e.g., acculturative stress and acculturation gap) in relation to suicidal ideation, planning, attempts, and/or deaths.

**Fig. 1:**
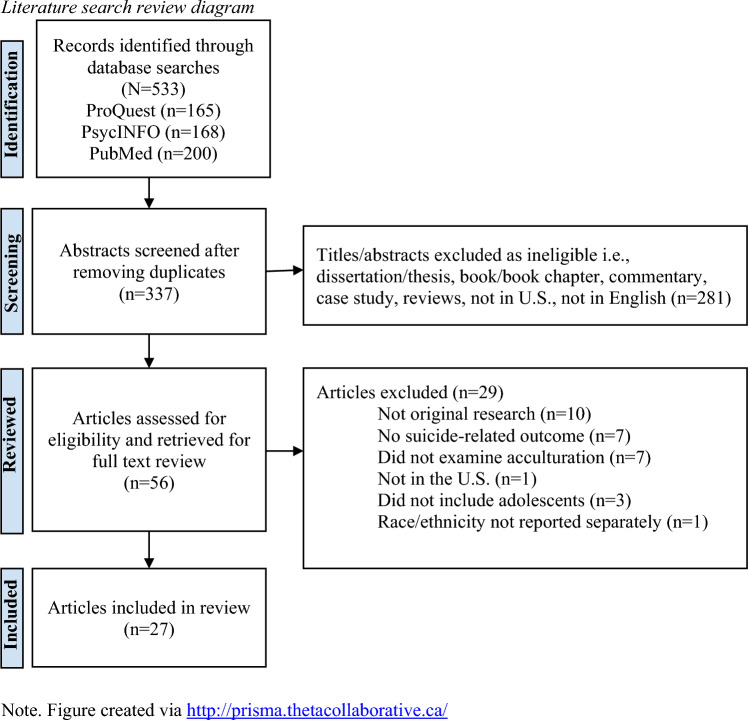
Literature search review diagram

Based on these inclusion criteria, 56 articles were initially identified; a full-text review yielded 27 articles for data extraction and coding (see Fig. 1 for further details on the selection process).

### Data extraction and coding

Included articles were reviewed and data were extracted and coded by four coauthors with graduate-level training in psychology or related fields. Each full text was independently reviewed and coded by two coauthors. Discrepancies were resolved by consensus or review by a third coauthor. The first and fourth coauthors developed the coding procedure and trained all coders for this study to extract and code the data from the included articles. Over 90% agreement was achieved across all coders with three randomly selected articles before proceeding to code the complete dataset. Data extraction collected descriptive information on the study sample and study characteristics of each article: sample size and demographics, theoretical frameworks used to conceptualize and operationalize acculturation and suicide risk, and whether the hypotheses were supported.

During the coding phase, the procedure focused on five coding-unit categories defined a priori: *theoretical perspectives* (e.g., Did article cite a theoretical framework? A suicide framework? Was/were the framework(s) named?); *study population and sampling methods* (e.g., What was the analytic sample size? Age range? Mean age? Breakdown by sex/gender, race/ethnicity, sexual orientation, etc.? Migration/generation status of the youth and/or parent?); *study design and analytic strategies* (e.g., What geographic location was the sample recruited from? Was it a school-based setting? Clinical setting? Community setting? Did the study use a quantitative, qualitative, or mixed-methods design?); *suicide assessment methods* (e.g., Were suicidal ideation, attempt, and/or death examined separately? Was suicide risk assessed with a single item and/or validated tool? Timeframe of the suicide-related outcome?); *assessment of acculturation* [Was acculturation assessed using demographic proxies? With a validated tool? Was a bidimensional framing used? What domains (i.e., practices, values, identity) were examined? Were acculturation-related constructs examined?]; and *main and other relevant findings* (e.g., Was a positive, negative, or no association found between acculturation-related and suicide-related constructs? What were the reported limitations? Was discrimination examined? Racial and/or ethnic identity? Racial and/or ethnic socialization?). The research team met weekly to review the codes, revise the coding method, and reconcile any discrepancies.

The risk of bias was coded with a tool for assessing study quality in cross-sectional and longitudinal cohorts, the Newcastle–Ottawa Scale (NOS) [41], which uses a star-rating scale. A star is assigned to procedures meeting high scientific rigor in participant selection (4 items), comparability of variables under investigation (1 item), and ascertainment of outcome variables (3 items). Eight stars indicated low risk for bias, 4–7 stars medium risk, and 0–3 stars high risk. This study was exempt from review by our institutional ethics board as it did not directly involve human participants.

### Data analysis

Due to significant heterogeneity in study methodologies, a quantitative meta-analytic approach was not appropriate for this review. Content analysis was used to synthesize the study findings to identify themes and gaps in the empirical literature. We used a directed approach—as our coding strategy was theory-driven and informed by existing empirical evidence—and updated it as new data emerged throughout coding [14]. The findings of the selected articles were first examined to identify the sample characteristics, recruitment strategies, study designs, and theoretical frameworks. Next, suicide-specific scales and acculturation-related measures were tallied to determine the scales operationalizing and assessing the constructs. Finally, associations of acculturation and acculturation-related constructs with suicidal thoughts and behaviors were identified and summarized. A “positive” association was found when greater acculturation to Euro-American society was associated with higher suicide-related risk; a “negative” association when greater acculturation to Euro-American society was associated with lower suicide-related risk; and a “null” association when the two constructs were not significantly associated.

## Results

### Narrative synthesis

Five themes emerged from the content analysis: (1) limited representativity in sampling, relying on purposive or convenience methods; (2) concerns regarding the quality of acculturation and suicide-related assessments; (3) stronger evidence for the impact of acculturative stress and other acculturation-related constructs on suicide-related risk than for acculturation itself; (4) overrepresentation of risk factors (deficit-model approach) and underrepresentation of protective factors (strengths-based approach); and (5) sparse attention to intersectional approaches and racialized experiences.

### Sample characteristics

Overall characteristics of the 27 articles included in the review are described in Table 1 and individual article characteristics in Table 2. Sixteen unique studies were represented with 1 article each, but the remaining 9 articles were included in the analysis because they used secondary data analyses to answer a different research question. The sample size ranged from under 100 to over 5,000; mean ages were 12.8–17.9 years, although five articles (18%) did not report mean age for the whole sample. Age ranges at inclusion, when available, were 10–20 years. Whereas nearly a quarter of the studies used national data, the remaining samples were based largely in the Northeast (including 10 from New York City), and the South.

**Table 1:** Characteristics of the 27 articles included in the review

	# (%) of articles
**Year of publication**	
2005–2010	5 (19%)
2011–2015	11 (41%)
2016–2022	11 (41%)
**Study design**	
Quantitative (cross-sectional)	16 (59%)
Quantitative (longitudinal)	6 (22%)
Qualitative/mixed method	5 (19%)
**Sampling strategy**	
School-based	17 (63%)
Community-based	10 (37%)
Clinic-based	11 (41%)
**Sample size**	
*n* < 100	6 (22%)
*n* = 100–500	7 (26%)
*n* = 500–1000	5 (19%)
*n* = 1000–5000	6 (22%)
*n* > 5000	3 (11%)
**Geographic region**	
National (mainland US)	6 (22%)
Northeast	13 (48%)
South	8 (30%)
West	4 (15%)
Midwest	2 (7%)
US territory/Alaska/Hawaii	1 (4%)
**Racial/ethnic groups**	
Latinx/Hispanic	14 (52%)
Black/African-American	1 (4%)
Asian/Pacific Islander/Native Hawaiian	3 (11%)
Middle Eastern/North African	1 (4%)
Ethnoracially diverse*	8 (30%)
**Acculturation assessment**	
Scales (multiple domains)	4 (15%)
Scales (single domain)	4 (15%)
Demographic as proxy	14 (52%)
Acculturation-related constructs^+^	7 (26%)
**Suicide risk assessment**	
Single-item question	15 (56%)
Semi-structured interview	10 (37%)
Self-report scales	2 (7%)
**Suicide outcomes examined**	
Suicide risk (unspecified)	1 (4%)
Suicide ideation/thoughts	12 (44%)
Suicide plans	2 (7%)
Suicide attempts/behaviors	15 (56%)
Suicide deaths	0

*The sample was inclusive of adolescents from a variety of racial/ethnic groups

^+^Includes acculturative stress, intergenerational conflict, and acculturation gap

**Table 2:** Characteristics by article

Authors	Sampling/demographics	Acculturation construct(s)/measure(s)	Suicide risk constructs/measure	Acculturation and suicide risk findings (+ = Positive; – = Negative; X = Null)	Risk of publication bias
Quantitative (cross-sectional)					
Peña et al. [5]	School (*N* = 3135) *M*_age_: 15.5 Gender: 49% F Race/ethnicity: 100% H/L	Proxies: generational status, Place of birth	Past Year SA: “During the past 12 months, how many times did you actually attempt suicide?”	( +) First-generation youth had lower rates of SA compared with second- and later-generation youth	Low
Joe et al. [7]	Community (*N* = 1170) *M*_age_: 15 Gender: 50% F Race/ethnicity: 69% BA, 31% CB	Proxies: generational status, adolescent and parent place of birth	Lifetime & Past year SI/SA: Composite International Diagnostic Interview (CIDI) [87]	( +) AA adolescents were five times more likely than CB youth to endorse SA; no difference was observed in SI	Low
Zayas et al. [68]	Clinical/community (*N* = 140) *M*_age_ = 15.2 Gender: 100% F Race/ethnicity: 100% H/L	Acculturation-practices (*Bidimensional Acculturation Scale for Hispanics*) [51]; acculturation-values (*Attitudinal Familism Scale*) [55]	Eligibility criteria: lifetime history of SA (diagnostic interview)	(X) Neither Hispanic orientation nor Anglo orientation in values and practices were associated with SA. Adolescents with SA history reported less mutuality with their mothers; mothers of adolescents with SA history also reported less mutuality	Low
Baumann et al. [67]	Clinical/community (*N* = 169) *M*_age_: 15.2 Gender: 100% F Race/ethnicity: 100% H/L	Acculturation-practices (*Bidimensional Acculturation Scale*) [51]; Acculturation-Values (Attitudinal Familism Scale) [55]	Eligibility criteria: lifetime history of SA (diagnostic interview)	(X) Acculturation gap (i.e., differences in Hispanic values), was directly associated with daughter-mother mutuality and externalizing behaviors, not daughter SA status	Low
Cho and Haslam, [69]	School (*N* = 227) *M*_age_: 16.6 Gender: 57% F Race/ethnicity: 100% A	Acculturation (Suinn-Lew Asian Self-Identity Acculturation Scale*)* [49]; Acculturation Stress (Social, Attitudinal, Familial, and Environmental Acculturative Stress Scale for Children; [88]); Length of time in US	Past month SI: Suicide Ideation Questionnaire (SIQ) [48]	( +) Neither Asian nor Western orientation levels in practices, values or identity were related to SI; acculturative stress was positively associated with SI, though not a significant predictor after adjusting for general life stress	Medium
Borges et al. [62]	School (*N* = 1004) Grades 9–12 Gender: 55% F US-Born 60–62% F Foreign-Born Parent(s) Race/ethnicity: 19–44% H/L, 40–52% B, 6–18% O, 3–27% W	Proxies: place of birth; years living in the US; parent birthplace; language practices	Past Year SI: ‘‘In the past 12 months, did you ever seriously consider attempting suicide?’’	(X)Language spoken at home, or length of residency in the US was not associated with SI; US-born (though not immigrant) youth reporting discrimination due to their own/family nativity had higher odds of SI	Low
Zayas et al. [63]	Clinical/community (*N* = 232) *M*_age_: 15.5 Gender: 100% F Race/ethnicity: 100% H/L	Acculturation-practices (Bidimensional acculturation scale) [51]	Eligibility criteria: past 6-month SA (diagnostic interview)	(X) SA status was not associated with Hispanic or US cultural involvement. Higher Hispanic cultural involvement was associated with greater mother–daughter mutuality, which in turn, was associated with lower risk of SA	Low
Cervantes et al. [71]	Clinical/School (*N* = 1254) *M*_age_: 14.9 Gender: 54% F Race/ethnicity: 33% H/L, 33% W, 21% O, 7% MX, 3% NA, 3% B	Acculturation stress (Hispanic Stress Inventory-Adolescents) [71]	Past 2 weeks SI: Child Depression Inventory-2 (CDI-2) [89]	( +) Acculturation-gap stress was associated with SI among females. Discrimination stress was associated with SI in males	Low
Piña-Watson et al. [73]	School (*N* = 345) *M*_age_: 15.71 Gender: 100% F Race/ethnicity: 100% H/L	Proxy: place of birth	Past year SI: ‘‘During the past 12 months, did you ever seriously think about committing suicide?’’	( +) Adjusting for depression symptoms, academic interest, parental caring, US birthplace increased odds of SI, though not among youth with both parents present	Low
Haboush-Deloye et al. [64]	Clinical/School (*N* = 103) *M*_age_: 15.39 Gender: NR Race/ethnicity: 51% W, 28% B, 18% H/L, 1% NA, 1% Jewish	Acculturation-Identity (General Ethnicity Questionnaire; [54]); Acculturation stress (Social, Attitudinal, Familial, and Environmental Acculturative Stress Scale for Children; [90])	Eligibility criteria: endorsed active SI during screening	( +) Acculturative stress was associated with SI and SA; Hispanics reported highest levels of acculturative stress; racial/ethnic identity predicted acculturative stress in youth at risk for SI	High
Piña-Watson et al. [61]	School (*N *= 516) *M*_age_: 16.23 Gender: 53% F Race/ethnicity: 100% H/L	Acculturation stress (Bicultural Stressors Scale) [91]; Proxy: Generational status	Past Year SI: “In the last 12 months, have you seriously thought about committing suicide?”	( +) Generational status was not associated with SI; bicultural stressors increased risk for SI	Low
Jones et al. [58]	School FL (*N* = 6212), Boston (*N* = 2314), NYC (*N* = 11,570), SF (*N* = 4374), Seattle (*N* = 1896) Grades 9–12 Gender: 48–50% F Race/ethnicity: 8–45% W, 9–40% B, 6–36% H/L, 2–49% A, 1–13% O	Proxies: place of birth; Years living in the US	Past Year SI: “In the last 12 months, have you seriously thought about committing suicide?”	(**–)** Immigrant youth reported greater SA than US-born youth; Fewer than 6 years in the US was associated with higher odds of SA for youth in FL, NYC, SF and Seattle; Among youth in Boston, greater than 6 years in the US was associated with higher odds of SA	Low
Hall et al. [74]	School (*N* = 7641) Grades 9–12 Gender: 53% F Race/ethnicity: 100% H/L	Proxies: place of birth, language practices	Past Year SA: “During the past 12 months, how many times did you actually attempt suicide?’’	(**–)** Higher odds of SA among foreign-born youth speaking language other than English at home, among girls not boys	Low
Piña-Watson et al. [72]	School (*N* = 722) *M*_age_: 17.89 Gender: 66% F Race/ethnicity: 100% H/L	Intergenerational acculturation conflict (Family Conflicts Scale) [92]	Past year suicide risk (unspecified): [93] “In the past year, have you thought about or attempted to kill yourself?”	( +) Intergenerational Acculturation Conflict (IAC) was significantly, positively correlated with suicide risk; caregiver connection attenuated the association between IAC and suicide risk	Medium
Roche et al. [60]	School/community ATL (*N* = 547) 55% F; *M*_age_: 12.8 DC (*N* = 340) 52% F; *M*_age_: 16.37 Race/ethnicity: 100% H/L	Proxies: generational status, place of birth, US residence, immigrant parents	Past 6-month SI: youth self-report [94] “I think about killing myself”	(X**)** Psychological/behavioral responses to immigration news was higher in youth with foreign-born (first-generation) vs. US-born (second-generation) parents, and was directly associated with increased odds of SI among both groups	Low
Stark, et al. [59]	School (*N* = 357) *M*_age_: 15.65 Gender: 47% F Race/ethnicity: NR	Proxies: place of birth, years living in the US	Past month SI: “if he or she reported having thoughts of ending his or her life never, sometimes, often, or almost always in the last month”	(**–)** Foreign-born status, particularly in the Middle Eastern/North African region, was associated with higher levels of SI	Low
Quantitative (longitudinal)					
Wong and Maffini [6]	School (*N* = 959) M_age_: 16.43 (W2) Gender: 48% F Race/ethnicity: 100% A	Proxies: place of birth; Language practices	Past year SI: “During the past 12 months, did you ever seriously think about committing suicide?” Past year SA: “During the past 12 months how many times did you actually attempt suicide?’’	( +) Greater orientation to American culture was positively correlated with SI, but not Wave 1 SA or Wave 2 SA; youth with greater American orientation had higher odds of having non-protective relationships, which was associated with higher odds of SA; having peer relationships was associated with SA risk for youth with lower orientation to American culture	Medium
Fried et al. [57]	School (*N* = 3376) *M*_age_: 14.86 (9th grade); 16.78 (11th grade); 51% F Race/ethnicity: 71% W, 12% O, 12% B, 5% H/L	Proxy: place of birth	Past year SA: ‘‘During the past 12 months how many times did you actually attempt suicide?’’	(+) US-born had higher odds of SA in the 9th grade but not in the 11th grade	Medium
Ortin et al. [70]	Clinical (*N* = 43) *M*_age_: 15 Gender: 81% F Race/ethnicity: 67% H/L, 16% B, 12% Mixed, 5% A	Acculturation and acculturation gap (Cultural Lifestyle Inventory) [50]	Past month SI: Suicide Ideation Questionnaire-Jr (SIQ) [48]	(+) Greater American and lower Hispanic orientation in adolescent (not parent) was associated with SI severity. Acculturation gap between adolescent and parent was not associated with SI, but emotion reactivity was increasingly negatively associated with SI as acculturation gap increased	High
Hishinuma et al. [65]	School (*N* = 2038) Ages: 14–17 Gender: 54% F Race/ethnicity: 30% NH, 13% W, 13% FA, 15% JA, 29% O	Acculturation-practices (Hawaiian Culture Scale-Adolescent) [52]	Past 6-month SA: Major Life Events Scale (MLES) [95]	(+) Native Hawaiian youth had higher SA rates than non-Native Hawaiian youth. Greater engagement in Native Hawaiian cultural practices did not predict SA	High
Choi et al. [66]	School/Community (W1-N = 786), *M*_age_: 15; (W2-*N* = 604), *M*_age_: 16.54; (W3-*N* = 641), *M*_age_: 18.06 Gender: 52% F R/e: 100% A	Intergenerational acculturation conflict (Family Conflicts Scale) [92]; Acculturation-Identity (Language, Identity, and Behavior Acculturation Scale) [53]	Past year SI: “if they seriously thought about committing suicide in the year prior to the survey”	( +) Intergenerational Acculturation Conflict (IAC) and racial discrimination was associated with greater odds of SI; level of ethnic identity was associated with lower odds of SI	Medium
Park and Park [56]	School (W1-*N* = 9787); (W2-*N* = 7553) Asian *M*_age_: 16.1, 53% M White *M*_age_: 16, 50% M Race/ethnicity: 85% W; 14% A	Proxies: place of birth; language	Past year SI: “During the past 12 months, did you ever seriously think about committing suicide?”	(+) Rates of SI were greater among US-born Asian-American youth who spoke English at home vs. foreign-born Asian-American youth who spoke a language other than English at home	Medium
Qualitative/mixed method					Not scored
Gulbas et al. [46]	Clinical/community (*N* = 73) *M*_age_: 15.3 Gender: 100% F Race/ethnicity: 100% H/L	Transnational stress; semi-structured interview	Eligibility criteria: lifetime history of SA (diagnostic interview)	(+) Nearly half of Latinas with SA expressed recurring themes of transnational stress	
Gulbas and Zayas, [43]	Clinical/community (*N* = 44) *M*_age_: 15.7 Gender: 100% F Race/ethnicity: 100% H/L	Acculturation (values); semi-structured interview	Eligibility criteria: lifetime history of SA (diagnostic interview)	(+) Themes of distress, interpersonal discord, and emotional isolation associated with SA history; compared to Latinas with SA history, those without were able to counter experiences of acculturative tension by creating supportive relationships with other individuals	
Humensky et al. [44]	Clinical/community (*N* = 31) Ages: 12–18 Gender: 100% F Race/ethnicity: 100% H/L	Acculturation gap; semi-structured interview	Eligibility criteria: in treatment for recent SA (diagnostic interview)	(+) Themes identified as risk factors for suicidal behaviors include conflict between youth and parents stemming from issues of acculturation, immigration, and gender roles; and struggles in school and with peer relationships	
Szlyk et al. [45]	Clinical/community (*N* = 33) *M*_age_: 15 Gender: 100% F Race/ethnicity: 100% H/L	Acculturation (practices, values), generational status, immigrant parents; semi-structured interview	Eligibility criteria: lifetime history of SA (diagnostic interview)	(+) Intergenerational dynamics within Latino families’ impact youth suicidal behavior	
Kennard et al. [42]	Clinical (*N* = 6 parent–child dyads) *M*_age_: 15.8 Gender: 83% F Race/ethnicity: 100% H/L	Acculturation (values), immigrant parents; semi-structured interview	Eligibility criteria: recent suicidal thoughts/behaviors (diagnostic interview)	(+) Familial and cultural dynamics, acculturation differences, and fears about discriminatory political and social environments impact Latinx youth and their families	

Race/ethnicity as reported in article. Age is reported as mean or range

*SI* suicide ideation, *SA* suicide attempt. For demographics: *A* Asian, *FA* Filipino American, *JA* Japanese American, *B* Black/African-American, *CB* Caribbean Black, *H/L* Hispanic/Latinx, *MX* Mixed, *NA* Native American, *NH* Native Hawaiian, *W* White/European-American, *O* Other; *F* Female, *M* Male, *NR* not reported

Most articles (*k* = 21; 78%) reported data collected via non-random (convenience or purposive) sampling methods. Over a third of the articles used more than one sampling method and recruited from multiple settings. Across the board, nearly two-thirds of the articles recruited from school settings, nearly half from clinical settings (e.g., emergency departments, inpatient/outpatient psychiatry units, and primary care), and a third from community-based services (e.g., social service agencies, after-school programs, and youth centers).

The majority (*k* = 19; 70%) of articles focused exclusively on a racially or ethnically minoritized group, largely Latinx adolescents, most of whom were of Mexican, Puerto Rican, Cuban, or Dominican descent; of these, 8 studies focused on Latina females. None of the articles that focused on Latinx adolescents reported on race, resulting in an unclear racial composition (e.g., White-Latinx, Afro-Latinx, and Indigenous-Latinx). Three articles (11%) focused exclusively on Asian-American adolescents, largely of Filipino, Korean, or Chinese background; one (4%) on African Americans and Black Caribbeans, though it was unclear whether Hispanic/Latinx youth were included in the latter group; and one (4%) on recently migrated adolescents from North Africa or the Middle East, which reported nationality but not race. All studies reported gender as binary (i.e., female/male). About half (*k* = 14; 52%) accounted for generation status in parent(s) and youth: first-generation migrants were defined as those born outside of the mainland US (not including territories) and second-generation migrants as those born in the mainland US to parents born abroad. Only three studies (11%) reported on gender identity/sexual orientation in the sample description (i.e., LGBTQ). None reported on religious identity.

### Study design and analytic strategies

Five articles used qualitative approaches [42–46] and employed thematic analysis to identify recurring themes from semi-structured interviews. The remaining articles employed quantitative approaches to collect survey data (*k* = 22; 81%), with more than half demonstrating low risk for bias on the NOS (*k* = 13/22; 59%), though about a quarter (*k* = 6/22; 27%) evidenced medium risk for bias and three (14%) evidenced high risk for bias. Most of the quantitative articles were cross-sectional (*k* = 16/22; 73%).

### Theoretical frameworks

Over three-fourths of all articles (*k* = 21; 78%) cited theoretical frameworks to guide their research questions and hypotheses; over half of these integrated multiple frameworks (*k* = 11/21; 52%). Over three-fourths of the articles that applied a theoretical framework focused on sociological, ecological, or cultural perspectives (*k* = 16/21; 76%), though no study used a racism-based framework. Suicide (*k* = 13/21; 62%) and developmental models (*k* = 10/21; 48%) were also cited. The most common suicide framework (*k* = 7; 33%) was the eco-developmental model of suicidal behaviors among Latinas [39]. The Interpersonal-Psychological Theory of Suicide (IPTS) [47] was also commonly referenced (*k* = 4; 19%), though this model was developed and largely applied in research for adults.

### Assessment of suicide risk

Single-item assessments were the most widely used measures of suicide risk outcomes and varied substantially in the wording of suicidal ideation (SI) questions, although less variability was detected in the wording of single-item questions assessing suicide attempts (SA) and plans. Only two articles used the self-report survey Suicidal Ideation Questionnaire-JR [48], one of the few suicide risk assessment tools that has demonstrated strong psychometric properties in ethnoracially minoritized youth. The rest used semi-structured diagnostic interviews. SA were the most widely assessed suicide-related outcome, followed by SI. Meanwhile, suicide plans were examined only in two articles and no articles assessed suicide deaths. One article measured suicide risk in general terms without specifying suicide-related outcomes. The most widely used timeframe for suicide-related outcomes was past year (*k* = 13; 48%) followed by lifetime (*k* = 8; 30%), with few studies examining past month (*k* = 3; 11%), past 6 months (*k* = 2; 7%), or past 2 weeks (*k* = 1; 4%).

### Assessment of acculturation and related variables

Among all articles (*k* = 27), more than half (*k* = 14; 52%) assessed acculturation using proxy demographic measures (e.g., nativity, generational status, and language proficiency). Nearly a third (*k* = 8; 30%) used a psychometrically validated self-report scale to assess acculturation; all independently assessed orientation toward heritage and mainstream culture, but none assessed heritage and mainstream orientation separately across all three domains of practices, values, and identity. Four (15%) articles assessed acculturation via multiple domains, including the Suinn-Lew Asian Self-Identity Acculturation Scale [49] and the Cultural Lifestyle Inventory [50]. Four studies (15%) examined acculturation via single domains. Specifically, articles examined acculturation via *practices* using the Bidimensional Acculturation Scale for Hispanics [51] or the Hawaiian Culture Scale-Adolescent [52]; via *identity* using the Language, Identity, and Behavior Acculturation Scale [53] or the General Ethnicity Questionnaire [54]; and via *values* using the Attitudinal Familism Scale [55]. The remaining five articles (19%), all qualitative studies, used open-ended semi-structured interviews. A quarter of the studies (*k* = 7; 26%) utilized acculturation-related constructs, typically those involving acculturative stress (*k* = 4; 15%; e.g., bicultural stress and transnational stress). Other acculturation-related constructs included acculturation gap—the discordance in acculturation levels between parents and children—and intergenerational conflict—the conflict within the parent–child dyad from differential acculturation.

### Main study findings

Overall, findings on the association between acculturation and suicide-related risk were mixed. Nineteen of 27 articles (70%) found a positive relation between acculturation or greater Euro-American orientation—assessed as demographic proxies, single/multiple domains, or acculturation-related constructs (e.g., acculturative stress and intergenerational conflict)—and rates of SI and/or SA. Three of the articles (11%) reported a negative association. Five (19%) reported no association. Most of the studies, however, were cross-sectional, relied on acculturation proxies such as demographic variables or acculturation-related constructs, used single-item assessments for suicide risk, relied on non-random sampling strategies, and did not account for racialized experiences.

There is some evidence that study findings were influenced by study characteristics, as all articles employing longitudinal designs reported a positive association, despite variability in their sample characteristics (e.g., size, recruitment setting, and race/ethnicity) and acculturation assessments. They also demonstrated medium-to-high risk for bias. Meanwhile, all articles reporting negative associations used cross-sectional designs and assessed acculturation using demographic variables as proxies; all demonstrated low risk for bias.

When acculturation was assessed via demographic proxies, findings were mixed. Five articles reported that greater acculturation to Euro-American society was associated with higher suicide-related risk in cross-sectional [5, 7] and longitudinal designs [6, 56, 57]. Five other articles, all cross-sectional studies, found higher risk among less-acculturated youth [58, 59] or no association at all [60–62]. All articles reporting on national samples (*k* = 6/27; 22%) also used demographic variables as proxies for acculturation and consistently reported a positive association between greater exposure to Euro-American society and higher rates of SI and SA; however, all these studies also used single-item assessments of SI and SA.

When acculturation was assessed bidimensionally (heritage and Euro-American) via single domains (practices, identity, or values), findings were also mixed. One study reported that increased heritage-culture practices and decreased Euro-American practices were associated with lower suicide-related risk [63]. However, one cross-sectional [64] and two longitudinal studies [65, 66] reported no association between heritage or mainstream cultural practices and suicide risk. When acculturation was assessed bidimensionally and across multiple domains, no association was found with suicide-related risk [60, 67–69], although these studies represent only two unique samples with a cross-sectional design. By contrast, one longitudinal study with largely Hispanic/Latinx adolescents reported that increased Euro-American practices and decreased heritage-culture practices were associated with higher severity of SI 1–3 months following discharge from the Emergency Department [70].

There was also evidence of some of the adverse effects of acculturation, as higher acculturative stress was consistently associated with higher risk for SI [43, 45– 48, 61, 64, 69, 71]. Of the four quantitative articles on acculturative stress, all except one [71] adjusted for levels of acculturation; however, each assessed it differently, as demographics proxies [61], as a single domain [64], or via multiple domains [69]. Similarly, one article found a positive association between acculturative stress and SA [64], adjusting for levels of acculturation via identity. Acculturation gap and intergenerational conflict in the context of acculturation gaps were also positively associated with elevated SI and/or SA in six articles [42, 44, 45, 66, 71, 72], although one article reported no association between acculturation gap and SI [70]. All the articles examining acculturation-related constructs, however, were cross-sectional, except for two that examined intergenerational conflict [66] and acculturation gap [70]. Four studies also examined discrimination due to nativity, ancestry, race, or ethnicity as a potential source of acculturation-related stress and found statistically significant positive associations [61, 62, 66, 71].

### Other relevant findings

Acculturation may differentially impact risk for SI and SA. One cross-sectional study reported a positive association with SA but not SI in a national sample of Black adolescents [7], whereas a longitudinal study with a national sample of Asian-American adolescents reported a positive association with SI but not SA [6].

Findings also suggested that the effects of acculturation on suicide-related risk may be influenced by family dynamics such as mother-daughter mutuality [63, 67, 68] and single- versus dual-parent household [73]. Acculturation-related differences between adolescent girls and their caregivers emerged in the context of gender-role conflicts [44, 45, 66, 72]. Moreover, the association of acculturation and related factors with suicide-related risk varied by gender [71, 74]. One article with ethnoracially diverse adolescents found that acculturation gap-related stress statistically predicted SI in girls, whereas discrimination stress statistically predicted SI in boys [71]. Differences in geographic location were also reported [58, 60]. For instance, in an ethnoracially diverse group of high school students, higher odds of SA were associated with less than 6 years in the US in Florida, New York City, San Francisco, and Seattle, and with more than 6 years in the US in Boston [58]. Despite reporting higher rates of SA in youth from sexual minority groups [57], no study examined acculturation differences in suicide-related risk by gender identity, sexual orientation, or other marginalized social identities.

## Discussion

Based on a content analysis of 27 research articles reporting on 16 unique studies published in English-language, peer-reviewed journals in 2005–2022, our scoping review of the empirical evidence finds a complex association between acculturation and suicide-related risk among US ethnoracially minoritized adolescents. Acculturative stress—including intergenerational conflict and experiences of racial/ethnic discrimination—and not acculturation itself, appears to be associated with greater suicide-related risk. The findings, however, are tempered by methodological shortcomings, including use of demographic proxies or single-domain assessments of acculturation, single-item assessments of suicidal thoughts and behaviors, cross-sectional study designs, and non-random, convenience sampling. Nevertheless, NOS findings indicate that over two-thirds of the reviewed articles evidenced low-to-medium risk for bias.

The evidence suggests that the association between acculturative stress and suicide-related risk may differ by gender, in part due to intergenerational conflicts exacerbated by differential acculturation regarding gender socialization. Adolescent females in contexts of more restrictive gender norms may be at elevated risk for SI compared to their peers raised following less-restrictive norms [75]. In addition, parental exposures to chronic stressors, including acculturative stress, may impact youth development and health-risk behaviors, particularly among ethnoracially minoritized youth [76]. Further investigation is warranted on the potential effect of acculturative stress across the lifespan and across generations [77]. Moreover, existing research over-emphasizes the study of risk factors rather than resilience, which went relatively unexamined. Future research should focus on factors that confer protection against suicide-related outcomes by fostering resilience [16–18].

The importance of acculturative stress detected in this review is consistent with earlier reviews on mental health outcomes in migrant groups that highlighted the critical role of stressful aspects of the acculturation process [25–28], which may be informed by the literature on racism as a social determinant of health [38]. Unfortunately, racism and racialized experiences were largely overlooked across frameworks and methodologies. For instance, although racial or ethnic differences were not detected in the association between acculturation and suicide risk, the generalizability of the findings remains unclear, as Latinx youth, particularly of Puerto Rican, Dominican, or Mexican background, were over-represented in study sampling. None of these articles, however, reported on race or skin color, which has been linked to health outcomes and remains understudied in health research among Latinx populations [78]. Although a few studies examined racial and ethnic identity and discrimination, no studies disaggregated race from ethnicity, or examined racial and ethnic socialization, or messages exchanged between caregiver and youth regarding racialized experiences [79], which are common in the lives of US ethnoracially minoritized youth [16–18]. Contemporary acculturation models recommend incorporating intersectional frameworks to capture the converging influence of multiple social identities on acculturative stress, including racialized experiences [32–34].

Contemporary models of acculturation also propose a dynamic, bidimensional process across multiple domains (though they overlook the centrality of acculturative stress, particularly as it relates to health), and only five articles used this operationalization. It is worth highlighting the findings of research with Latinx youth using this operationalization with longitudinal designs to study risk for substance use. These studies identified complex and more nuanced interactions between acculturation and acculturative stress that may inform suicide-related risk among ethnoracially minoritized youth [80]; however, these studies are similarly limited in their attention to racism and other racialized experiences.

Greater consideration of developmental [34] and ecological frameworks beyond family systems [18] in the acculturative process would also advance this area of study. For instance, whereas most studies focused on intrapersonal and interpersonal effects of acculturation, particularly within the family context, less is known about the impact on acculturation-related processes of macro-level systems at the school, neighborhood, institutional, or societal level. Another important consideration that is missing from the reviewed studies is how acculturative processes manifest in the digital age, especially as ethnoracially minoritized youth spend a substantial amount of time online [81]. Growing evidence indicates that social media use among youth may impact acculturation processes [34]. This is important to examine, given evidence that social media use has been linked with SI and SA risk in youth [82].

Findings are also tempered by limitations in the assessment of suicide risk, as studies relied largely on single-item assessments, which may not be reliable [83]. Future research should use psychometrically strong suicide risk assessment tools demonstrating reliability and validity in ethnoracially minoritized adolescents such as the Self-Injurious Thoughts and Behaviors Interview [84, 85]. Such research should also examine the differential impact of acculturative experiences on how it may uniquely confer suicide-related risk across the spectrum from SI to SA to suicide deaths. This distinction would help identify novel targets of intervention to better meet the mental health needs of migrant and ethnoracially minoritized youth in the US tailored to varying degrees of risk for suicidality.

### Limitations

Scoping review methodologies face inherent limitations regarding the appraisal of study design and methodology, lack of synthesis of the data, and broad search strategies and inclusion/exclusion criteria that may miss relevant articles [86]. For instance, we only included English-language, peer-reviewed empirical papers published in 2005–2022; a wider search might yield different findings. The global implications of the findings are not clear as all studies included were based in the US Due to variability in study design, sampling, and assessments, a meta-analysis was not conducted. Although 27 articles were included in the review, only 16 unique studies are represented, which also hindered quantitative analysis of the findings. Lastly, the significant heterogeneity within ethnoracially minoritized youth is obscured by their categorization as a single group. Despite these limitations, our findings highlight important evidence of the role of acculturation stress and suicide-related risk in ethnoracially minoritized youth in the US Importantly, we identify critical gaps in the literature and suggest next steps to clarify the impact of acculturation-related experiences on suicide-related risk among migrant and ethnoracially minoritized adolescents.

## Conclusions

The increasing ethnoracial diversity of US youth and the disproportionate increase in suicide risk among ethnoracially minoritized adolescents call for clarification of the potential role of acculturation in suicidal thoughts and behaviors. Findings from this review indicate that acculturative stress may confer risk for suicidal thoughts and behaviors in this population, and that the relation between acculturation more generally and suicidality is complex and warrants further examination, particularly with respect to social determinants of health, including racialized experiences. Specifically, acculturative stress resulting from family conflicts and racial/ethnic discrimination may play a larger role than acculturation per se in influencing suicide-related risk among migrant and ethnoracially minoritized youth. A developmental approach and systematic application of an intersectional research framework that accounts for racialized experiences would broaden our understanding of the mechanisms by which acculturation shapes risk of suicidal thoughts and behavior. This research is necessary to promote more culturally responsive youth suicide-prevention strategies.

## Data Availability

The data are available upon request. Please direct all requests to the lead author.
